# Adapt and conquer: Metabolic flexibility in cancer growth, invasion and evasion

**DOI:** 10.1016/j.molmet.2019.08.021

**Published:** 2019-10-10

**Authors:** Peter Kreuzaler, Yulia Panina, Joanna Segal, Mariia Yuneva

**Affiliations:** The Francis Crick Institute, London, UK

**Keywords:** Tumourigenesis, Tumour metabolism, Metabolic flexibility, Central carbon metabolism

## Abstract

**Background:**

It has been known for close to a century that, on average, tumors have a metabolism that is different from those found in healthy tissues. Typically, tumors show a biosynthetic metabolism that distinguishes itself by engaging in large scale aerobic glycolysis, heightened flux through the pentose phosphate pathway, and increased glutaminolysis among other means. However, it is becoming equally clear that non tumorous tissues at times can engage in similar metabolism, while tumors show a high degree of metabolic flexibility reacting to cues, and stresses in their local environment.

**Scope of the review:**

In this review, we want to scrutinize historic and recent research on metabolism, comparing and contrasting oncogenic and physiological metabolic states. This will allow us to better define states of *bona fide* tumor metabolism. We will further contextualize the stress response and the metabolic evolutionary trajectory seen in tumors, and how these contribute to tumor progression. Lastly, we will analyze the implications of these characteristics with respect to therapy response.

**Major conclusions:**

In our review, we argue that there is not one single oncogenic state, but rather a diverse set of oncogenic states. These are grounded on a physiological proliferative/wound healing program but distinguish themselves due to their large scale of proliferation, mutations, and transcriptional changes in key metabolic pathways, and the adaptations to widespread stress signals within tumors. We find evidence for the necessity of metabolic flexibility and stress responses in tumor progression and how these responses in turn shape oncogenic progression. Lastly, we find evidence for the notion that the metabolic adaptability of tumors frequently frustrates therapeutic interventions.

## 1 Introduction

In 1924, Otto von Warburg reported his observation that tumors engage in the seemingly more wasteful metabolic state of aerobic glycolysis, with lactate as an end product, while the analogous healthy tissues carry out complete oxidation of glucose to carbon dioxide via oxidative phosphorylation. This simplified view of oncogenic versus normal physiological metabolism remains the main textbook knowledge of cancer metabolism to date and is commonly known as the Warburg effect [1].

However, both historic data and newer research show that what is regarded as cancerous metabolism is not as clear-cut as previously thought. Different tissues and even specialized cells within a tissue can have unique metabolic properties. Moreover, periods of special energetic needs result in further unique metabolic phenotypes, such as during embryonic development or wound healing. This array of metabotypes are produced by differences in flux rates and flux ratios through varying metabolic pathways. All these pathways are available to cancer cells to utilize for their own benefit, which they do depending on the tissue context in which they grow or the stage of the disease - initiation, progression, and metastatic dissemination. This creates a level of flexibility that questions the assumption that there is a single, defining, static cancer metabolism.

In this review we explore the metabolic adaptations enabled by inherent flexibility of metabolism involved in cancer development, progression, and response to local stresses and therapy. We highlight the importance of understanding the diverse factors influencing changes in cancer metabolism and the potentially therapeutically targetable metabolic vulnerabilities that these changes create.

## 2 Metabolic changes from healthy tissues to neoplasia

Almost one hundred years from Otto von Warburg's initial observation relating to cancer metabolism, and particularly in recent years, it is fair to say that more effort has been directed towards understanding pathological metabolism than its healthy counterpart. To date, it is assumed that tumors require an extensive metabolic adaptation to grow and spread. Renewed interest in gaining a more holistic view of metabolism has led to some seminal studies in both normal and cancerous metabolism. By comparing the available data for both cancer and normal tissue metabolism, we tackle the question of whether or not there is such a thing as a cancer-specific metabolism, or if tumors merely hijack proliferative programs seen in normal tissues.

The central carbon metabolism is the backbone of cellular energy fluxes and, as such, lends itself well to compare oncogenic and physiological metabolism. One of the common features of neoplastic tissues is energetically and metabolically seemingly wasteful aerobic glycolysis, resulting in lactate production [2]. Equally, a general propensity for increased glucose intake by cancers compared to surrounding tissues is undisputed and forms the basis of some of the most powerful diagnostic tools such as FDG-PET [3]. However, large scale lactate production from glucose has been recognized in healthy tissues and usually coincides with cell division or highly energy-demanding processes. For example, the gut, one of the most actively proliferating tissues in adult mammals, has long been known as a net lactate producer, indicative of aerobic glycolysis [4,5]. Embryogenesis is equally marked by widespread proliferation, coinciding with large scale aerobic glycolysis [6,7]. Interestingly, other proliferative tissues such as skin, the mammary gland during gestation, and the juvenile heart during the postnatal phase of intense cell division, as well as wounds during the healing process have been linked to increased local lactate production under aerobic conditions [[8], [9], [10], [11]]. Other aspects of presumed tumor specific metabolism, such as increased pentose phosphate pathway (PPP) and uptake of glutamine and other amino acids, are beyond the scope of this review to be discussed in detail but have been observed in proliferative tissues and wounds [[12], [13], [14], [15], [16]]. Collectively, these features are part of a biosynthetic metabolism, in which carbon units are maintained as biological building blocks, rather than being fully oxidized for energy production. This type of metabolism is engaged by all proliferating tissues in physiology and pathology [17].

Thus, at face value, tumor tissues might not look that metabolically different from normal proliferative tissues. This is somewhat unsurprising, as the majority of identified driver mutations in cancers play an analogous role in normal proliferative processes, and virtually all of these have a direct involvement in metabolic modulation [18,19]. For example, the oncogene c-Myc (Myc), which is rarely mutated, but one of the most frequently amplified or afferently activated transcription factors in cancer [20], is responsible for direct transcriptional upregulation of a number of glycolytic genes and orchestrates the aforementioned biosynthetic metabolism [[21], [22], [23], [24]]. Myc is equally known to increase a tumor's reliance on glutamine as a source for energy and biosynthesis [25] and to upregulate PPP [26] as well as the Krebs cycle, although the latter two are co-regulated by the oxygenation status of the tumor [27,28].

There are a number of other pathways that are typically engaged downstream of growth stimuli and that frequently coordinate with Myc, which are recurrently mutated in cancers, but also found in areas of physiological proliferation. The most prominent are Ras [[29], [30], [31]] and the PI3K/AKT/mTOR axis [32]. Overexpression of these pathways leads to similar metabolic states as the one commandeered by Myc, and can be generally classified as biosynthetic with aerobic glycolysis, glutaminolysis, increased PPP and lipid biosynthesis among others [19,28,33,34]. It is worth noting that, while many aspects of the programs downstream of different oncogenes overlap, they are affected both by the primary driver oncogene and by environmental cues. For example, Ras-driven lung tumor cells in culture show a stark dependence on glutamine, while the same is not true *in vivo* [35]. This dependence has been shown to resurface in conjunction with a Keap-1 mutation, highlighting the context specificity of metabolic dependencies in an overall similar biosynthetic context [36]. Interestingly, even common oncogenes, such as chromatin binding protein polybromo 1 (PBRM1), that do not seem to lie directly on any of these pathways, have been found to profoundly influence cellular metabolism, tilting it towards biosynthesis [37]. Other transcription factors, such as BACH1, have been shown to reduce the activity of the electron transport chain (ETC) in triple negative breast cancers, thus reducing oxidative phosphorylation. Inhibition of BACH1 re-engages the ETC and increases cellular reliance on this pathway, and consequently sensitizing these tumors to metformin treatment, an inhibitor of the ETC [38].

While many *bona fide* oncogenes converge towards a biosynthetic metabolism, which includes aerobic glycolysis, tumor suppressors tend to do the opposite. Probably the most studied molecule in this regard is p53, a major stress sensor and the most commonly mutated or lost tumor suppressor in cancers. Among its wide-reaching activities is the switch from a Warburg-like proliferative metabolism to a less glycolytic metabolism [[39], [40], [41], [42], [43]]. Losing p53 thus further engrains a Warburg-like metabolism into cancerous tissues. Interestingly, mutations in p53 can give the protein neomorphic properties, and some of these mutations seem to actively influence tumorigenesis by affecting the metabolism. Indeed, a recent study showed that the R27 mutant version of p53 increases PGC-1α activity compared to the WT control, leading to increased mitochondrial biogenesis, epithelial to mesenchymal transition (EMT), and metastatic spread [44].

So far it would seem that tumors merely hijack well-defined metabolic pathways for their purposes by activating oncogenes and inhibiting tumor suppressors, both of which have prominent roles in regulating overarching metabolic pathways. However, the sum of these pathways seems to differ in normal tissues and tumors. For example, lactate, the original identifier for cancerous metabolism, reaches levels in tumors (up to 40 mM and more) that are higher than in any normal tissue under physiological conditions (1–5 mM depending on tissues) or even wounds (5–15 mM) [2,11,[45], [46], [47], [48]]. The individual cell producing or consuming lactate at any given stage during tumorigenesis might not be different from individual cells engaged in this type of metabolism elsewhere in the body, but under physiological conditions, production and consumption are maintained in a dynamic equilibrium, while in cancers this equilibrium is skewed and leads to net accumulation of lactate and other end products within the tumor. This is most likely due to the sheer proportion of proliferating cells, the widespread presence of hypoxic regions (see below), and the further systemic handling of the resulting lactate.

Under physiological conditions, the classic example for large scale lactate production is the Cori cycle, which has been known since the 1930s and describes a process in which, under stringent exercise, muscles produce lactate, which is used in the liver for gluconeogenesis [49]. Such carbon sharing turns lactate from a waste product to a transporter of carbons and highly energetic bonds. Indeed, lactate has a plasma concentration of about 1 mM even in resting individuals and, as such, represents the second largest blood bound carbon pool [50]. Consequently, recent research using elegant methods of infusions with stable isotope labeled glucose and lactate and calculating their steady state fluxes has concluded that a substantial proportion of glucose is delivered to healthy tissues in the form of lactate. Strikingly, with the notable exception of the brain, glucose derived carbon delivery to every tissue studied could be largely accounted for by lactate [51]. It is important to note that some of these results have come under intense scrutiny. The aforementioned experiments unambiguously show that lactate carbons can be funneled into the Krebs cycle, but they cannot give sufficient insights into the net consumption and production of lactate, as well as the amount of isotopic exchange that can take place between lactate and pyruvate, which can lead to an overestimation of the lactate contribution [52,53]. Indeed, these more recent data seem to tip the balance back towards glucose as the main contributor to the Krebs cycle. While this matter is not settled and might indeed be context dependent, aerobic glycolysis and subsequent carbon transfer may be more widespread than previously anticipated.

On a much smaller scale, carbon sharing via lactate appears likely to occur between cells of the same organ. The best studied example is found in the brain, where astrocytes are believed to funnel lactate to neurons after aerobic glycolysis [[54], [55], [56], [57], [58]]. Similarly, a symbiotic relationship has been suggested in organoid cultures between Lgr5-positive intestinal stem cells and the surrounding paneth cells, where the latter are thought to engage in aerobic glycolysis and pass carbons in the form of lactate to the stem cells, which need this type of metabolism for correct differentiation [59,60].

Interestingly, lactate is not shunned by tumors as a source of carbons. This is shown in recently published seminal work in human tumors, which proves that cancerous masses can quite avidly consume lactate, their own presumed waste product, to meet their energetic demands [61] and that metabolite consumption is heavily dependent on the originating tissue [62]. The widespread accumulation of lactate in tumors, however, indicates that overall there is a net production of lactate. A pressing issue to fully understand these observations, would be to reveal on a cellular basis, if there are producers and consumers of lactate in one tumor mass, with the latter outweighing the former, or if indeed individual cells engage in both production and consumption, with the former outweighing the latter.

One of the main differences between cancer metabolism and its healthy counterpart thus is not the underlying program engaged, but rather a lack of regulation thereof. The most common oncogenic drivers engage cancer cells in a continuous proliferative state and, in the same way, trap tumor cells in a metabolic “always on” state. This prevents cancer cells from reacting to exogenous and endogenous cues such as nutrient abundance, oxygenation, perfusion, redox state, and acidification in the same way that normal tissues would (discussed below). It is the sum of the individual components of this exaggerated and persistent state of activation that is idiosyncratic for tumor metabolism. The results of this metabolism are indeed so dramatic that at later stages of cancer development the effect can become systemic, as seen by the appearance of lactic acidosis, ketosis, and cachexia [63,64].

We have so far explored tumor metabolism driven by aberrant engagement of major metabolic genes priming tumors for certain hard-wired metabolic programs. More recent work, however, has discovered that many tumors engage in metabolic processes that are unique to tumor cells. These reactions frequently arise from a gain of function mutation that enables enzymes to produce novel compounds, or the loss of an enzyme that leads to an accumulation of compounds that normally only exist at low levels. These so called oncometabolites have sparked a lot of interest as they can be used as diagnostic markers or possibly even as therapeutic targets. This is particularly interesting as some oncometabolites have been shown to carry active roles in tumor progression and possibly even tumor initiation [65,66].

Probably the most prominent oncometabolite is (R)-2-hydroxyglutarate (2-HG), produced by mutated forms of isocitrate-dehydrogenase (IDH) 1 and 2, which preferentially reduce 2-ketoglutarate to 2-HG [67]. This metabolite is usually only present in vanishing amounts, but its concentration increases significantly in tumors, in which it interferes with a number of pathways such as hypoxia induced factor (HIF) activation or histone methylation [68]. Importantly, it was found that 2-HG can inhibit the transaminases Bcat1 and 2, thus affecting branched chain amino acid turnover and reducing the amount of glutamate produced from these amino acids. This precludes a major source of glutamate, and consequently cells with mutant IDH proved to be exceptionally sensitive to inhibition of glutaminase by CB-839, as this reaction represents the second major source of glutamate [69,70]. Interestingly, Myc overexpression in breast cancer can lead to 2-HG accumulation even without an IDH mutation due to a striking increase in glutaminase, which can funnel glutamine into this pathway [71].

Other oncometabolites include intermediates of the Krebs cycle, such as succinate and fumarate, which accumulate due to mutations in the enzymes that usually turn them over, namely succinate dehydrogenase and fumarate hydratase. Mutation of the former has wide reaching effects including increased reliance on pyruvate carboxylation via pyruvate carboxylase, thus potentially exposing a vulnerability [72]. It has a number of other consequences, such as impairing HIF signaling and leading to CpG hypermethylation [73,74], which markedly influence the tumor transcriptome and metabolome. Accumulation of fumarate also has a plethora of activities. It has been shown to promote EMT by inhibiting antimetastatic miRNA cluster *mir-200ba429* [75], activate non-canonical NF-kB signaling [76], and inactivate the mTOR pathway [77]. Understanding the underlying biology of these oncometabolites is starting to allow tailor made interventions [65].

Taken together, contrasting Warburg's original hypothesis, cancer specific metabolism seems to be based on a physiological proliferative metabolic program. However, in its exaggerated form, it does show marked deviations from metabolism under physiological conditions and can thus be classified as a distinct metabolic state, and an adaptation that covers the metabolic needs of a continuously growing cellular mass, while eventually enabling it to invade and spread. The more recent discovery of true oncometabolites, which arise from loss or mutations of metabolic enzymes, has further vindicated Warburg's idea of metabolism that is unique to cancer. This metabolism, however, is not consistent between all cancers, as tumors differ significantly in their metabolic wiring, depending on the underlying mutational landscape, the tissue of origin and the microenvironment.

## 3 Local metabolic adaptation

So far, we have discussed how genetic changes in tumors lead to a metabolic state that is derived from normal proliferative and regenerative programs, albeit being different from them in its net outcome. Individual tumors themselves, however, show quite striking differences in their regional metabolism, mostly due to differences in the microenvironment, and the supply of nutrients and oxygen. In the following section, we will shine a light on the major pathways of acute tumoral stress adaptations and how they interplay with oncogenic drivers and shape tumor evolution while potentially exposing vulnerabilities.

**Figure 1:**
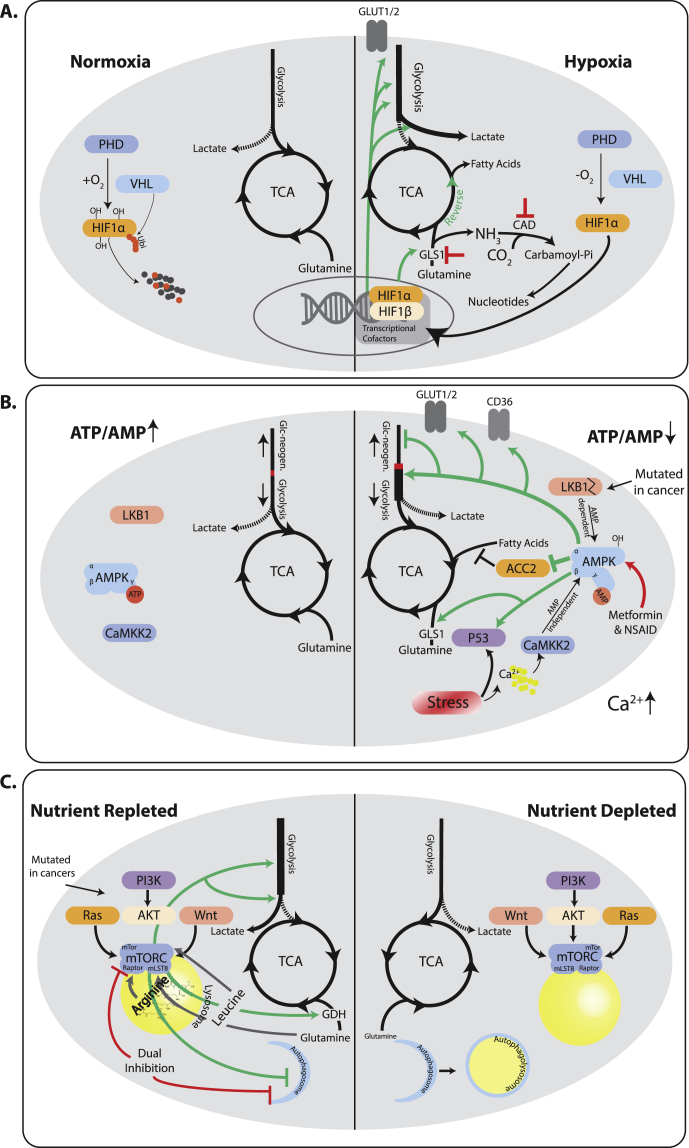
**Metabolic adaptations to acute metabolic stresses in the tumor microenvironment changes central carbon metabolism flow and exposes vulnerabilities**. A) Under normoxic conditions proline residues on HIF1ɑ are hydroxylated by PHDs, thus creating recognition sites for VHL, which ubiquitinates HIF1ɑ marking it for proteasomal degradation. Lack of oxygen prevents hydroxylation and degradation. HIF1ɑ stabilization leads to activation of a transcriptional program that increased glycolytic flux and shunts pyruvate towards glucose. Glutamine is funneled into the Krebs cycle and frequently used for fatty acid synthesis through reductive flux. Due to the higher turnover of glutamine, targeting GLS1 or CAD are trialed as therapeutic approaches. The former precludes a source of nutrients, the latter leads to an accumulation of ammonia. B) A high ATP/AMP ratio leads to binding of ATP to AMPK thus rendering it inactive. Growth stimuli and oncogenes can engage growth programs unabatedly. A drop of this ratio, leads to AMP binding to AMPK, which allows LKB1 to phosphorylate and activate it. AMPK can quench biosynthetic pathways and engage energy production, by boosting nutrient utilization in the Krebs cycle, including glucose, glutamine, and fatty acids, as well as nutrient uptake via transporters. LKB1 is frequently mutated in lung and cervical cancers, leading to failed engagement of LKB1. Persistent stress can, however, re-engage AMPK via a Calcium dependent AMP independent pathway carried out by CaMKK2. Thus, tumors frequently retain parts of this stress response pathway. Drugs including metformin and non-steroidal anti-inflammatory drugs (NSAID) have been shown to re-engage AMPK as well and are in clinical trial as therapies. C) The mTOR complex needs signals both from afferent proliferative pathways as well as nutrient sensors to be fully active. Once fully active, it engages a biosynthetic Warburg like metabolism with enhanced Glycolysis, pyruvate shunting into lactate, and increased glutaminolysis. mTor strongly inhibits autophagy, and inhibition of mTor induces autophagy leading to cell survival. Dual inhibition of mTor and autophagy is a promising drug combination. Note that some upstream activators can have mTor independent effects on metabolism that are not depicted. mTor is rarely mutated itself, allowing tumors to sense and react to nutrient depletion in tumors. When mTor is inhibited due to lack of nutrients, metabolism becomes more oxidative and autophagy is engaged, until amino acid levels have been replenished.

In healthy vertebrates, cells live under largely homeostatic conditions for most of the time. Nonetheless, they have a plethora of stress response pathways that allow them to mitigate the effects of environmental fluctuations, such as those seen in wounds, disease, or during extreme fasting and exercise [78,79]. This toolbox is at the disposal of tumor cells as well, and the activation of most stress response pathways in tumors proves that they make ample use of it. In fact, the cancer specific metabolism discussed above is frequently the net result of the underlying landscape of driving oncogenes and the metabolic changes that tumor cells undergo during stress responses. Thus, they are very much two faces of the same coin (Figure 1).

Tumor cells face pressure from two sides. On the one hand, at least a proportion of cells in the tumor are stuck in a proliferative loop that they cannot readily drop out of, while concomitantly being unable to generate sufficient microenvironment to support such growth. This is most evident in the lack of a homogenous blood supply throughout tumors, which leads to hypoxia, but also over-acidification, nutrient paucity and ultimately necrosis, with all its inflammatory side-effects [[80], [81], [82], [83]]. As discussed later, an interesting twist to this aspect is that while poor vascularization is a substantial initial challenge, some tumor cells adapt to survive in these regions and become difficult to treat due to a lack of drug delivery.

A lack of adequate vascularization bestows a number of metabolic impasses on tumors, the most acute of which is a lack of oxygen. Some tumors can have large part of their mass in a hypoxic state, but ultimately survive thanks to the activity of adaptive stress responses. Most of these stress responses pivot on the activity of a family of oxygen sensing transcription factors called HIFs, and its two major components HIF1ɑ, Hif2ɑ. The full breadth of HIF activities has been reviewed elsewhere (e.g., [84]), and we will focus on the most central activities. HIF proteins are generally unstable under oxygenated conditions, due to the activity of prolyl hydroxylases (PHD), which hydroxylate prolines on HIF, which are in turn recognized by the Von Hippel–Lindau (VHL) complex, ubiquitinated and degraded [85,86]. Lack of oxygen prevents hydroxylation and thus stabilizes HIF. Once stabilized, HIFs exert profound activities on the central carbon metabolic pathways. They increases intake of glucose, glycolytic flux, for example, through upregulation of hexokinase 2 (HK2), and lactate production, diverging carbons away from the Krebs cycle and thus contributing to lactate accumulation in tumors [27,84,87]. Conversely, HIF stabilization also increases the uptake of glutamine and its conversion to ɑ-ketoglutarate, which is then frequently used in a reductive Krebs cycle for conversion into citrate and fatty acid synthesis [88]. Lastly, HIF counter-balances the hypoxic state, by inducing vascular endothelial growth factor (VEGF) and thus stimulating angiogenesis [89]. Interestingly, it has been shown that HIF and the oncogene Myc act synergistically in parts of this stress response, including increased expression of HK2 and VEGF [22]. This exemplifies how the net outcome in tumor metabolism can be a joint activity between driver oncogenes and stress response genes.

The question naturally arises whether HIFs are *bona fide* oncogenes themselves. It is known that loss of VHL leads to increased levels of HIF1ɑ and in a more tissue restricted manner HIF2ɑ, and that this predisposes to tumors such as clear cell renal cell carcinoma (ccRCC) [90,91]. More recent studies seem to imply that the activities of HIF1ɑ and HIF2ɑ might indeed be antagonistic in renal carcinomas. Specific inhibitors of HIF2ɑ showed a marked therapeutic effect, although it seemed to hinge on an active p53 pathways, while targeting HIF1ɑ did not show the same therapeutic effects [92]. Indeed, it appears that HIF1ɑ might even act as a tumor suppressor under these circumstances, highlighting a currently poorly understood interaction between the two transcription factors [93,94]. These observations are consistent with reports that Hif1ɑ, at least under certain circumstances, antagonizes the activity of the oncogene Myc, while HIF2ɑ synergizes with it [95,96]. Furthermore, stabilization of HIF by competitive inhibition of PHD through accumulation of fumarate in FH mutant tumors is associated with hereditary leiomyomatosis and renal cell carcinoma (HLRCC), although it is unclear if this is a bystander effect or a requirement [97,98]. This notwithstanding, amplifications or stabilizing mutations of both Hif1ɑ and Hif2ɑ are rare in human tumors. This might be due to tumor suppressive pathways, which are induced by HIFs after prolonged hypoxia. Thus, while the relative contributions of the individual HIF family members still need to be fully disentangled, it is almost certainly advantageous for the tumor to retain the ability of reverting back to a normoxic metabolism, when oxygen is present.

Independently of the trigger for HIF1ɑ accumulation, these tumor cells acquire metabolic states with seemingly reduced flexibility. It was recently shown that glutaminase 1 (GLS1), when induced by hypoxia, is required for tumor progression. This pathway becomes so relevant that inhibition thereof significantly suppresses metastasis and cell migration [99]. Other systems have been reported to have a remarkable ability to adapt to therapies targeted at glutaminolysis; it would thus be interesting to compare the flexibility of those same systems under more restrained circumstances such as hypoxia [100]. Another problem facing hypoxic cells is the disposal of the glutamine nitrogen without causing a toxic accumulation of ammonia. This is in part accomplished by conversion of glutamine derived carbamoyl-Pi into dihydroorotate by the enzyme dihydroorotase (CAD). This pathway is crucial for survival of cells in this metabolic state, as knockdown of CAD leads to tumor shrinkage [101]. The stress response to hypoxia thus enables cells to persist under adverse conditions, and shapes the net tumor metabolism, but also creates synthetic lethality, which would not affect normoxic cells (Figure 1). Long term adaptation to these conditions, however, seems to push tumor evolution towards more aggressive and metabolically adaptive phenotypes, which are involved in tumor progression and resistance, as will be discussed below.

Another consequence of the poorly vascularized, but hyperproliferative tumor environment is frequent nutrient scarcity. As expected, cells are equipped with a machinery to sense and react to such states of nutrient deprivation, and tumor cells can make use of them, thus yet again adopting a stress-response type of metabolism. Interestingly, tumors have a somewhat ambivalent relationship with these pathways, as they typically act as potent growth suppressors as well. The ability to react to nutrient deprivation is thus counterbalanced by the overall proliferative advantage of tumor clones that have disengaged from such constraints, but might now be more likely to run into an energetic catastrophe. Clones managing to perform these balancing acts are thought to have enhanced adaptability, which hampers targeted therapies.

The best defined rheostat for cellular energy levels is the serine/threonine kinase AMPK (activating AMP-activated protein kinase). This kinase is activated by the binding of AMP, which signals a low energetic status of the cells [102,103]. When activated, AMPK phosphorylates a number of targets that tilt the metabolic state of the cell from anabolic to catabolic [104]. This includes increased uptake of glucose as well as fatty acids, increased glycolytic flux and a shift towards non-glucose carbon utilization in the Krebs cycle, including glutamine [[105], [106], [107], [108], [109], [110], [111], [112]]. On the other end of the metabolic spectrum, AMPK inhibits gluconeogenesis via phosphorylating CRTC2 and inhibits glycogen storage via inhibition of glycogen synthases, GYS1 and GYS2 [113,114]. AMPK has even been shown to activate p53 either via direct phosphorylation or more likely via the inhibition of its inhibitor MDMX [115,116], thus activating the ample tumor suppressive downstream program governed by p53.

The AMPK activities mentioned so far would suggest that AMPK is a *bona fide* tumor suppressor. This is underpinned by studies showing that loss of AMPK can indeed promote tumor growth in leukemias [117,118]. However in human tumors, it is not AMPK that is frequently lost, but rather its upstream activator LKB1, whose phosphorylation of AMP bound AMPK at Thr^172^ is required for full AMPK activation. LKB1 is mutated in up to 35% of non-small cell lung cancer (NSCLC) and 20% of cervical carcinomas, making it the dominant tumor suppressor gene in this signaling axis affected in tumorigenesis [119,120]. Originally, this was believed to be due to the fact that LKB1 could activate a whole family of related kinases, and its loss would prevent a global inhibition of downstream pathways [121]. However, more recent data have challenged this idea, and it has become clear that retaining some AMPK activity in tumors can be beneficial, as shown in breast and lung tumors [122,123]. In the latter case, AMPK increased survival of tumor cells by upregulating lysosomal biogenesis, and possibly autophagy as a means of keeping tumor cells alive. It is worth noting that it has been shown that even in the absence of LKB1, AMPK can be activated by alternative regulators, such as CAMKK2, which reacts to increased cellular calcium flux following stress signals [124]. This allows a more restricted activation of AMPK, which might strike the balance between sustaining growth even under adverse conditions, but avoiding cell death when cellular stress becomes more severe. Recent evidence that AMPK can be activated by a number of drugs such as metformin and non-steroidal anti-inflammatory drugs (NSAIDs) sparked attempts to use its tumor suppressive activity in tumor therapy (Figure 1) [125].

While AMPK is central to the detection of energetic stress, another cellular complex, termed mTOR, is the central integrator of growth factors on the one hand and cellular nutrient status on the other hand (for an extensive review [32]). mTOR is usually kept in check by another complex, termed TSC [126]. A number of growth factors, such as IGF-1, via AKT activation, but also receptor tyrosine kinases, such as EGFR, via Ras, as well as Wnt, ultimately lead to phosphorylation and consequently inactivation of the TSC complex, thus allowing mTOR to signal in principle [[127], [128], [129]]. Furthermore, recent finding have shown a growth factor independent pathway in which Ras mediated enhanced lactate production interferes with the TSC mediated mTOR inhibition [130]. For a full activation of mTOR, however, certain prerequisites need to be met. In particular, mTOR activity is reliant on a number of lysosome-bound complexes, which only allow full activation when they sense an abundance of their cognate amino acids such as lysine and arginine [32,[131], [132], [133], [134]]. Other signals of metabolic stress equally bear down on mTOR, such as via AMPK, which can phosphorylate and inactivate mTOR, as well as other less well defined sensors of glucose availability or p53 following DNA damage [135,136]. mTOR is fully activated and engages a large anabolic downstream program only when these sensors signal sufficient nutrient availability. At the same time, mTOR activity inhibits catabolic processes, such as protein turnover and autophagy [32,137,138].

In particular the inhibition of autophagy by mTOR makes energetic sense, as mTOR is active when lysosomes contain sufficient amino acids, and further cellular degradation is thus unnecessary. Conversely, a constitutively active mTOR complex would prevent autophagy in tumor cells under nutrient depletion, thus depriving them of a well-documented source of energy provision under such circumstances. This might explain why mTOR is frequently hyperactivated by upstream effectors such as constitutive AKT signaling, loss of PTEN or Ras mutation, but rarely through mutations that lead to constitutive activation [139,140]. This is another example of how tumors might need to strike a balance between growing in an inhospitable environment on the one hand, all the while retaining the ability to react, if the stresses become too severe.

As reflected in its name, mTOR was found as the target of the anti-proliferative compound Rapamycin. It was thus regarded a promising drug target as soon as it was found, but it has since been plagued by a number of clinical setbacks. One of the reasons is that inhibition of mTOR frequently leads to induction of autophagy [141]. While autophagy has been proposed to be both oncogenic and tumor suppressive, in a context dependent manner [142], it seems to be mostly pro-survival in established tumors. Dual inhibition of mTOR and autophagy has, however, shown promise in pre-clinical models and is now in trial in the clinic (Figure 1) [[143], [144], [145], [146], [147]].

A consequence of the high metabolic activity and unstable blood supply in tumors is an increased production of reactive oxygen species (ROS) such as O_2_^−^, H_2_O_2_, and OH^−^, mainly due to electron leakage from ETC as well as the NADPH oxidases (NOXs) [148,149]. While these compounds have long been believed to be mere toxic waste products, it is now clear that they play a role in both physiological processes as well as in oncogenic transformation and progression [150].

ROS species have been shown to inhibit the activity of growth inhibiting phosphatases [151,152] and to be instrumental in the growth of some tumors such as KRAS driven lung cancer [152]. Importantly, ROS can also be involved in the early stages of tumorigenesis itself or its progression, as evidenced by the predisposition to tumors in patients with mutations in the succinate-ubiquinone oxidoreductase subunit D gene (SDHD), or the metastatic progression of cells harboring mutations in NADH dehydrogenase subunit 6 (ND6) [[153], [154], [155]]. These mutations increase the cellular ROS burden due to defects in ETC components, which, in turn, is believed to increase the mutational incidence.

These activities are counterbalanced by the clearly deleterious effects of excessive cellular ROS which activate potent tumor suppressive pathways and can incapacitate the cell's ability to function due to oxidation of its proteins, DNA, and lipids. Cells thus possess powerful antioxidant programs. The most prominent one is the glutathione system. Reduced glutathione is synthesized from cysteine, glutamate, and glycine. Glutathione peroxidase converts H_2_O_2_ to water and oxidized glutathione. The latter can be converted back to reduced glutathione by glutathione reductase and NADPH [156,157]. An alternative system is the thioredoxin system, in which cytosolic or mitochondrial peroxiredoxins can convert H_2_O_2_ to water by oxidizing their active site. Thioredoxin reductase (TrxR), and NADPH can recover reduced peroxiredoxins [158]. Besides its biosynthetic activities NADPH thus plays an important role in ROS management as well.

Interestingly, tumors frequently upregulate both the production and pools of ROS, as well as the antioxidant program [159,160]. This seems to allow to harness some of the beneficial effect of ROS on cancer biology, while keeping ROS levels in check when they reach dangerous levels. The central rheostat for this type of cellular stress is constituted by the Kelch-like ECH-associated protein 1 (Keap1) - nuclear factor erythroid 2 like 2 (NRF2) system. Keap1 is a negative regulator of Nrf2, which, in turn, is a transcription factor that will enhance transcription of a coordinated antioxidant pathway [161,162]. Keap1 can be oxidized, when ROS levels increase, which, in turn, prevents NRF2 from proteasomal degradation and initiates the antioxidative program. It is thus unsurprising, that Keap 1 is frequently mutated in tumors, while Nrf2 can undergo stabilizing mutations [19,163]. Engagement of the antioxidant response has a knock-on effect on the central carbon metabolism as it subtracts large amounts of glutamine from the Krebs cycle funneling them into glutathione production [164]. This effect is exacerbated by the need to import cystine, equally essential for glutathione production, which is in large part accomplished via the SLC7A11/xCT cystine/glutamate antiporter [165]. Cancer therapies targeting the dependence of tumors upon a working antioxidative machinery and the resulting effects on carbon metabolism are thus in trial and have shown some promise [36,164,166,167].

Taken together, cancer cells make use of a number of well-established stress responses that are hard wired into mammalian cells. A common theme in their adaptation to the harsh environment within tumors, and to retain a significant proliferation, is that the tight regulation on important metabolic aspects such as energy status and nutrient availability is somewhat loosened. Ultimately, however, it seems advantageous to retain some form of stress response, as the ability to occasionally drop out of cycle, when the overall conditions become too taxing, will lend an overall growth advantage, and can promote metastatic progression.

## 4 Metabolic adaptation during metastatic progression

As discussed above, cancer cells are able to adapt their metabolism in response to environmental stresses in the primary tumor. However, stress responses also have been shown to coincide with the instigation of metastasis and poor prognosis, and metastatic disease remains the leading cause of mortality in cancer patients [[168], [169], [170]]. Metastasis involves tumor cells spreading to adjacent healthy tissue [171], undergoing EMT that allows them to enter the vasculature [172], and reverting back to an epithelial-like state (MET) [173] as they colonize distant organs. During this process, tumor cells migrate through highly diverse environments with different oxygen and nutrient availability, suggesting a fundamental requirement of metabolic adaptations for survival. Furthermore, metabolic enzymes and metabolites are known to have direct signaling functions, which contribute to distinct metabolic reprogramming at each stage of the metastatic cascade.

A major topic of debate is whether all cells in the primary tumor are able to metastasize when faced with certain environmental stresses, or if a small subset of cells predisposed to metastasis exists in the primary tumor. Wagenblast et al. demonstrated that subclones in the primary tumor expressing specific anticoagulant proteins are able to metastasize [174]. In another study, Kang et al. identified four specific genes that allow breast cancer cells to metastasize to the bone, and subclones in the primary tumor that express these genes were highly metastatic [175]. Using barcoding tracking to evaluating cloning dynamics of breast cancer metastases to different organs revealed that certain clones dominate metastases in different organs [176]. These findings suggest that a certain genetic predisposition is important for primary cells to be able to metastasize to specific organs. Importantly, cancer stem cells have been proposed as a metabolically flexible sub-population of the tumor that contributes to metastasis (further reviewed in [177]), for example in breast [178] and colorectal [179] cancers. A combination of mutational background as well as the acquisition of permissive cellular states is thus most likely required for cells to spread.

**Figure 2:**
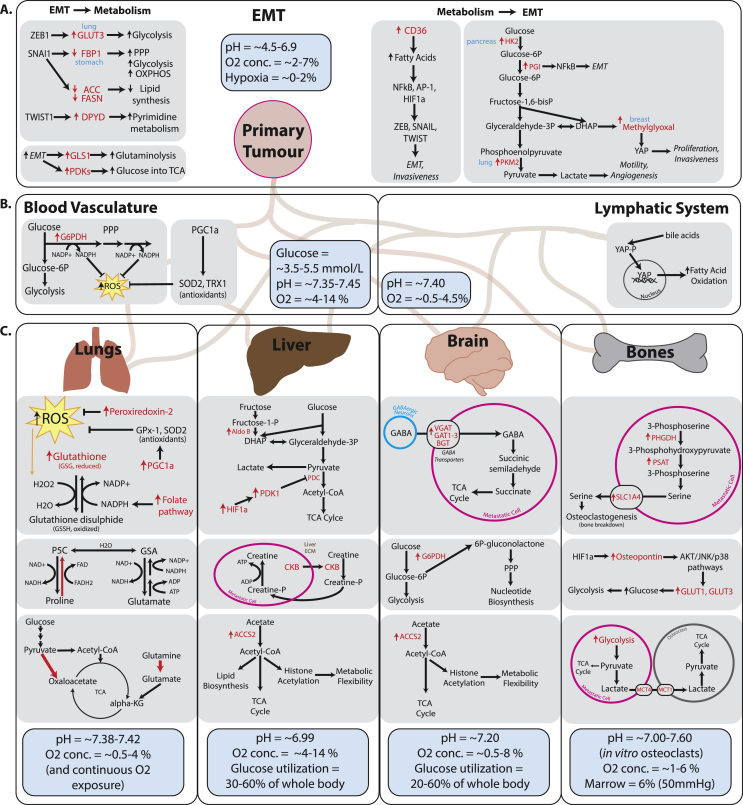
**Metabolic adaptations contribute to every step of metastasis progression.** The physiological pH, oxygen concentration, and notable nutrient availability in each organ are summarized in blue boxes. A. Metabolic adaptations in the primary tumor during EMT and metastasis instigation. EMT transcription factors have been shown to directly signal to metabolic enzymes (left), and in turn, metabolic transformations can also directly upregulate pro-EMT signaling (right). B. Metabolic adaptations in blood vasculature and the lymphatic system. Circulating tumor cells are faced with increased oxygen availability and therefore upregulate anti-ROS pathways. In the lymph, a novel role for bile acid metabolism has been shown to promote fatty acid oxidation via YAP activation. C. Differential metabolic adaptations observed in metastases to the four most common sites of dissemination: lungs, liver, brain and bones. The metabolic adaptations shown here have been observed in models of different cancer types however the metabolic environment of the organ being colonized may influence metabolic adaptations of metastases through similar mechanisms. Due to continuous oxygen exposure, metastases to the lung upregulate several different mechanisms to combat oxidative stress and promote oxidative phosphorylation for energy and biomass production. Liver metastases make use of the availability of alternative carbon sources by upregulating fructose and acetate metabolism, and exploit the host organ's extracellular matrix for synthesizing creatine phosphate, which then contributes to ATP production in the metastasis cells. Metastases to the brain also adapt to use alternative available fuel sources including acetate and GABA and also promote PPP for nucleotide biosynthesis. Finally, metastases to the bone adapt their metabolism to help them exploit the natural bone-breaking osteoclast cells in order to make more room for metastasis expansion. It has been shown that bone metastasis cells excrete serine and lactate to promote osteoclastogenesis. Furthermore, bone metastases excrete the bone-specific signaling protein osteopontin which upregulates glucose intake for glycolysis. Figure 2 references: Oxygen concentrations in healthy tissues [263], in tumors [264]. Glucose concentrations [265,266], in blood [267], utilization by liver [268], utilization by brain [269,270]. Intratissue pH [[271], [272], [273]], intratumor pH [274], bone pH [273], bone marrow pH [275].

Although EMT is not an absolute requirement for metastasis [180], it typically plays a major role in priming cancer cells to be able to spread to distal organs [181]. Transcription factors driving EMT can directly signal to metabolic pathways to support the bioenergetic demands of increased motility and survival in new environments (Figure 2A). For example, the EMT-initiating transcription factor zinc finger E-box binding homeobox-1 (ZEB1) (the pathophysiological role of which is further reviewed in [182]) has been shown to directly upregulate the glucose transporter GLUT3, leading to increased glycolytic flux in NSCLC [183], and SNAI1 overexpression redirects carbons from glucose towards oxidative PPP by downregulating fructose-1,6-bisphosphatase (FBP1) expression in gastric cancer [184].

On the other hand, it has also been shown that changes in cancer cell intrinsic metabolism can directly drive EMT initiation (Figure 2A). Below, we will highlight how major metabolic pathways including glycolysis, lipid metabolism, and mitochondrial metabolism play a role in driving EMT. Upregulation of glycolytic enzymes HK2 and pyruvate kinase muscle isozyme M2 (PKM2) have been linked to increased motility and invasion, through upregulation of matrix metalloprotease 2 (MMP2) and VEGF [185,186]. Furthermore, activation of yes-associated protein (YAP) by the glycolytic byproduct methylglyoxal drives proliferation and invasiveness [187,188]. Increased lactate production supports chemoattraction and migration [189] and alters tumor pH, which directly instigates cell invasiveness [190] and leads to secretion of cathepsins and matrix metalloproteinases for ECM degradation via NFκB activation [191]. Another glycolytic enzyme, phosphoglucose isomerase (PGI) acts as a secreted cytokine to directly upregulate NFκB, which induces EMT by stabilizing ZEB1/2 [192]. Upregulation of the glycolytic enzymes aldolase-A and glyceraldehyde-3-phosphate dehydrogenase (GAPDH) was shown to be required for increased motility and EMT initiation via SNAIL upregulation in NSCLC [193,194].

EMT activation has been shown, in turn, to be driven by increased lipid metabolism in colorectal cancer, potentially by increasing expression of matrix metalloproteinases, maintaining the energetic demands of dissemination and activating the Akt/Erk pathways by maintaining a balance of signaling monounsaturated and saturated fatty acids [195]. Furthermore, upregulation of the fatty acid receptor CD36 in liver cancer also induced EMT through the Wnt pathway [196]. Signaling lipids have been shown to directly control cancer progression and dissemination. For example, neutrophil-derived leukotrienes promote selective breast cancer aggressiveness and prime the pre-metastatic niche in the lungs [197]. Another class of signaling lipids, epoxyeicosatrienoic acids (EETs) were shown to cause metastasis to several distal organs by increasing VEGF production and therefore angiogenesis [198]. The roles of signaling lipids in metastases have been reviewed in detail elsewhere [199].

Mutations in mitochondrial metabolic enzymes of the Krebs cycle have also been shown to directly drive EMT in different cancer types [75,200], and mitochondrial metabolism deregulation (further reviewed in [201]) has been linked to poor prognosis [202]. Finally, Knott et al. have shown that EMT and metastatic development are directly linked to the expression of asparagine synthetase and asparagine availability in breast cancer cells, since EMT-associated proteins were shown to have 19% higher asparagine content than total proteome percentage, and knock-down of ATF4, a transcriptional regulator of asparagine synthetase, leads to decreased EMT potential [203].

Most of the mentioned studies focus on a specific cancer subtype, so it is important to note that different mechanisms may play more significant roles depending on the site or driving oncogene of the primary tumor. Nevertheless, all of these findings suggest that metabolic rewiring plays an essential role in the initiation of metastasis through EMT.

As tumor cells intravasate into the vasculature they are faced with a drastic increase in oxygen availability and, therefore, reactive oxygen species. To cope with this stress, circulating tumor cells (CTCs) upregulate antioxidant pathways [204]. Furthermore, it has been shown that the ability to cope with oxidative stress may be essential in some cancer types, since folate pathway inhibition decreases the metastatic potential of melanoma cells [205], and treatment with the antioxidant N-acetylcysteine conversely promotes melanoma metastasis [206]. Similarly, metastatic breast cancer CTCs increase their antioxidant capacity by upregulating PPP metabolism to generate more NADPH, which is needed for glutathione recycling [207]. Upregulation of peroxisome proliferator-activated receptor gamma coactivator 1-alpha (PGC1a) was also seen in breast cancer CTCs, leading to increased oxidative phosphorylation and ROS balance [208] (Figure 2B).

Another factor influencing metabolic adaptations in metastasis is the differential preference of certain types of cancer to leave the primary tumor either via lymphatic or blood vessels [209]. For example, compared to blood vessels, lymphatic vessel formation has been shown to be directly dependent on fatty acid beta-oxidation with a lower dependency on glycolysis [210]. Furthermore, lymph node metastases upregulate YAP-mediated fatty acid oxidation for biomass production [211] (Figure 2B). Several studies have reported various physio-chemical factors that may dictate a preference for one or the other, such as hypoxia gradients, vessel density, tightness of endothelial cell junctions, and, importantly, the presence of specific signaling molecules that promote lymphangiogenesis or angiogenesis and act as chemoattractants (further reviewed in [[212], [213], [214]]).

Metastasizing cells may undergo metabolic rewiring when they enter and colonize a distal organ, either to support increased demand for ATP and biomass for proliferation, or as a result of differential nutrient and oxygen availability. An increasing number of studies have shown distinct metastatic metabolism to be instructed by the organ being colonized rather than the organ of origin of the primary tumor. We will therefore discuss the metabolic adaptations that tumor cells undergo when they enter and survive in specific distal sites (Figure 2C).

The lungs are one of the most common metastatic sites [215], meaning that cancer cells that survive there have to adapt to the lung's unique oxygen and nutrient availability. Breast cancer metastases to the lung have upregulated pyruvate carboxylase-dependent anaplerosis as a result of increased pyruvate availability [216]. Due to high oxygen availability, and therefore high ROS levels, lung metastases were shown to increase production of the antioxidative protein peroxiredoxin-2 [217], increase glutamine catabolism for oxidative phosphorylation [218], and increase proline metabolism to support FADH2 production for ATP production through the electron transport chain [219]. A study looking at melanoma metastatic potential demonstrated that distal metastases have increased levels of key enzymes in the folate pathway to produce NADPH for glutathione recycling, further shown by an increased NADPH/NADP ratio in metastases to the lung and other organs compared to that of the primary tumor [205]. Furthermore, the observed PGC1a upregulation and necessity in circulating tumor cells and metastases [220,221] may play a role in ROS level regulation, since PGC1a promotes expression of antioxidant genes GPx-1 and SOD2 [222].

On the other hand, liver metastases were shown to adapt to lower oxygen availability in certain regions of the liver by upregulating pyruvate dehydrogenase kinase (PDK-1) in a HIF-1a-dependent manner, promoting pyruvate conversion to lactate rather than the Krebs cycle [218]. Colorectal cancer liver metastases have been shown to secrete creatine kinase brain-type (CKB) into the ECM. CKB converts creatine to phosphocreatine, which is then taken up by metastatic cells, and the phosphate group transferred to ADP for ATP production [223]. Another study demonstrated that colorectal cancer liver metastases have upregulated aldolase B, the key enzyme for fructose metabolism, suggesting an adaptation that allows liver metastases to readily utilize fructose as an alternative carbon source [224].

Metastatic cells may adapt their metabolism to mimic that of the healthy cells around them to survive in the new environment. Metastases to the brain show extreme metabolic flexibility, mimicking neuronal metabolic plasticity. Like healthy brain cells, brain metastases are able to upregulate gluconeogenesis enzymes such as FBP1 in response to lower glucose availability and oxidize glutamine and branched chain amino acids for ATP and biomass production [225]. Furthermore, brain metastases have been shown to have increased activity of PPP supporting nucleotide biosynthesis [226] and increased GABA uptake and metabolism to fuel the Krebs cycle [227].

Similarly to maturing osteoclasts, breast cancer bone metastases have upregulated *de novo* serine production enzymes (PHGDH, PSAT1, PSPH) and the serine transporter SLC1A4 [228]. It has been proposed that metastatic cells can release excess serine, which was shown to be a requirement for osteoclastogenesis and bone breakdown to make space for metastatic expansion [229]. Furthermore, highly glycolytic bone metastases were observed to release excess lactate via the MCT4 transporter, and the lactate was subsequently taken up by osteoclasts through the MCT1 transporter [230]. Lactate could then be used to fuel osteoclast proliferation through oxidative phosphorylation. Finally, bone metastases express the osteoclast-specific glycoprotein osteopontin [231,232], which has been shown to be upregulated in a hypoxia-dependent manner and to promote cell adhesion as well as glucose uptake into osteosarcoma cells [233].

The emerging trend in recent studies reviewed above is that the organ being colonized may dictate metabolic adaptations in metastasis. Although, in some studies, factors like hypoxia, a specific nutrient availability, and an interaction with the cells forming a “metastatic niche” are suggested to induce these adaptations [234], in a lot of cases how these metabolic phenotypes are imposed is not known and a clonal selection of tumor cells with permissive metabolic qualities cannot be excluded. There are also other factors to consider when designing potential therapeutic strategies. Recently, it was reported that the metastatic burden, defined by the frequency and size of metastatic lesions, promotes differential gene expression in triple negative human breast cancer PDX models [235]. Lawson et al. found that low burden metastases have a more basal, dormant phenotype, while high burden metastases favor proliferation and cell cycle progression by upregulating Myc suggesting that there may be further metabolic changes as metastases progress.

To summarize, metabolic flexibility is required for cancer cell survival throughout the process of metastasis and fuels distinct survival mechanisms in different distal sites. The varying gradients of nutrient and oxygen availability produce stresses in specific cells within the primary tumor, driving them towards an EMT state and promoting metastasis. While the majority of recent studies suggest that environmental stress can directly trigger EMT, certain clonal populations in the primary tumor have also been shown to have a selective advantage in colonizing distant sites (metastasis initiating cells, MICs) [176]. Nevertheless, once metastasizing cells leave the primary tumor site, they absolutely must continuously adapt their metabolism to the distinct environment of the blood or lymph, and then of the new distal organ they colonize. The majority of the papers reviewed here focus on a particular cancer model and often do not assess the presence of CTCs or metastasis in all the organs of the body. While this presents a limitation to these studies, the diversity of findings reveal that cancer cells are able to deploy various genetic and metabolic programs in order to assure their survival in diverse environments. There are several points of consensus emerging, such as that of the role of ROS in circulation and the lung environment, the role of lipid metabolism in liver metastasis, and the crosstalk between genes involved in metabolism and EMT instigation. The cells that adapt accordingly and disseminate to distant organs successfully are most often responsible for cancer fatality. Therefore, it is vital to understand the diverse metabolic vulnerabilities that cancer cells develop during metastasis.

Future work needs to assess how findings in *in vitro* systems and animal models translate to human disease, and how metabolic vulnerabilities created in the process of metastasis can be targeted in combination with traditional chemotherapy and immunotherapy regimens. Importantly, several studies have shown that the mechanisms of metastatic resistance to treatment and relapse arise through metabolic adaptations.

## 5 Metabolic adaptation to treatment

So far we have discussed the flexibility of a tumor enabling it to respond to endogenous environments within the body in which it grows. However, tumors also require a degree of flexibility when their status quo is acutely perturbed through drug administration in order to resist treatment. Non-responsiveness to therapeutics and/or post-treatment relapse is an unresolved problem in oncology. This occurs due to poor predictive power of the likelihood of a patient to respond to a given treatment course, resistant clones within a tumor surviving treatment due to unique cellular properties of those clones, failure of drugs to reach all cells in the tumor to therapeutic levels leaving pockets of unaffected cells, and adaptation of tumor cells to specific targeted treatments. Metabolic rewiring associated with tumor formation and progression can affect all of the above. Therefore, understanding the metabolic changes associated with cancer treatments will enable more effective anti-tumor interventions.

**Figure 3:**
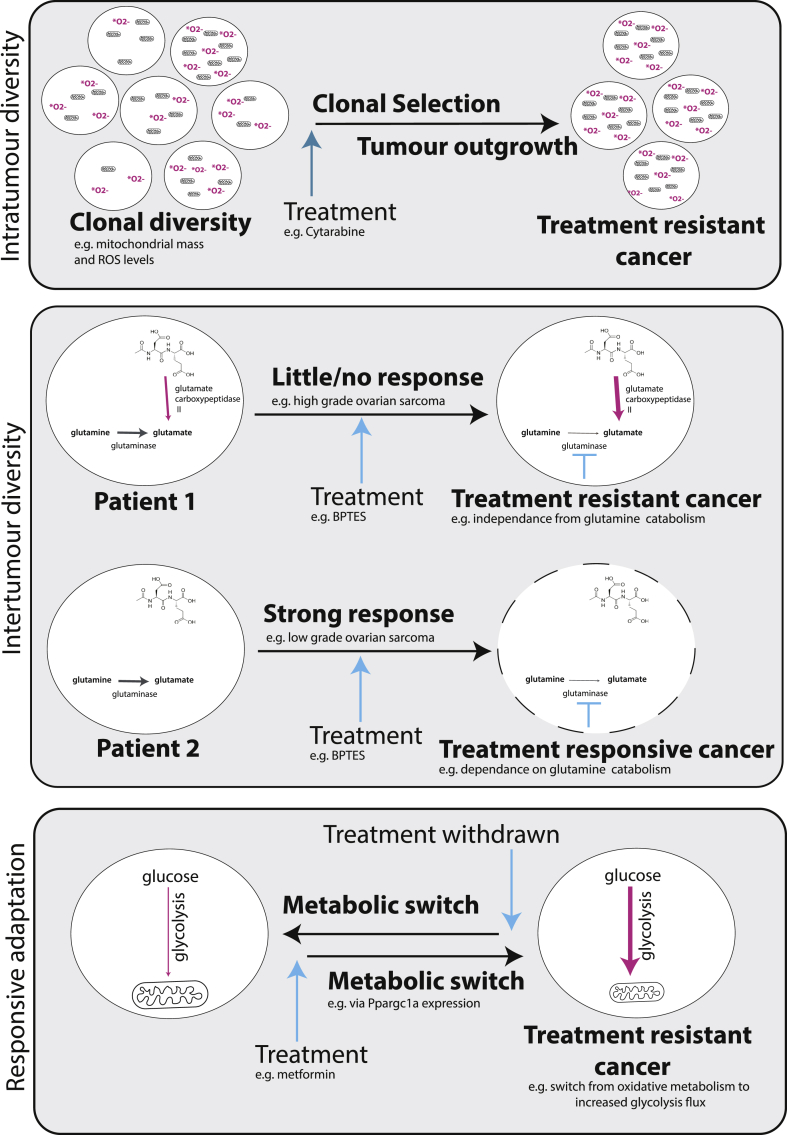
**Metabolic diversity and adaptability enable tumors to resist treatment.** Diversity may occur within a tumor, where treatment selects for clones most able to overcome treatment, or between tumors, resulting in differential responses between patients due to inherent metabolic differences. Tumor cells may also rewire their metabolism as a direct response to treatment to minimize the effect on the cell while retaining their ability to revert to their nascent metabolism when the treatment program ends.

It has been shown that metabolic differences can result in resistance of some tumors to treatments, including first-line chemotherapies. However, it is not well-established if this is a result of prior differences in the metabolism of these tumors or a subset of cells within the tumors, or whether this is a specific reactive change in response to treatment exposure (Figure 3).

An example of where prior metabolic differences likely affect treatment outcome stems from metabolically-induced differences in cell state. Classic chemotherapy confers specificity to tumor cells in large by targeting rapidly dividing cells. However, quiescent cells within the tumor cell population do exist. Quiescence can be induced through interrupted metabolic substrate supply [236,237] and acidosis caused by lactate accumulation [238,239]. These non-dividing or slow-cycling cells induced by extreme metabolic environments tolerate the chemotherapy and, following the end of a cycle of treatment, can go on to repopulate the tumor [240].

Metabolic differences between actively proliferating cells of the same tumor can also affect treatment outcome. Farge et al. showed that Cytarabine-resistance in AML is a result of a pre-existing subset of cells that persist following treatment. These cells displayed higher levels of reactive oxygen species and increased mitochondrial mass, consistent with a more oxidative phenotype compared to the cells which succumb to the chemotherapy [241]. In breast cancer it has been shown that the cancer stem cell population makes up the more oxidative, more resistant subset of cells in a tumor, which, in this study, was found to be driven by co-overexpression of Myc and MCL-1 [242]. Alternatively, whole tumors can be made up of more or less oxidative cells that result in cancers that are accordingly more or less resistant to treatment. For example, in a study of melanoma, cancers that showed more oxidative phenotypes prior to any treatment, resulted in a higher level of resistance to treatment when it was applied [243].

The expression of certain metabolic enzymes can also provide a subset of tumors with an advantage over particular treatments. The use of small molecule glutaminase inhibitors (targeting the reaction responsible for the conversion of glutamine to glutamate) as cancer treatments has reached the stage of clinical trials [244]. However, it has been shown that certain types of tumors can show resistance to this treatment through utilization of alternative metabolic pathways that enable cells to bypass the need for this reaction to maintain growth. One such example of the types of tumors that show a degree of resistance to the glutaminase inhibitor, BPTES, treatment is the case of high-grade ovarian serous adenocarcinoma, which, unlike low-grade tumors, have the capacity to breakdown N-acetyl-aspartyl-glutamate by expression of glutamate carboxypeptidase II. This enzyme enables the high-grade tumors to source glutamate independently from the glutaminase reaction [245].

Acquired resistance can also be induced as a consequence of treatment in the form of adaptive reprogramming. Brown et al. demonstrated the adaptation of breast cancer cells to genotoxic therapeutic agents [246]. Here, *de novo* biosynthesis of pyrimidine was enhanced in cells that were treated with genotoxic chemotherapy, cisplatin, compared to unexposed cells. This adaptive response was facilitated through metabolic regulation of the multifunctional enzyme responsible for the first three steps of pyrimidine synthesis, CAD - carbamoyl-phosphate synthetase 2, aspartate transcarbamoylase, dihydroorotase - and allowed the cells to maintain cell division to the rate seen in untreated cells. Similarly, in a study of metastatic breast cancer cells, Andrzejewski et al. demonstrated metabolic rewiring in cells that acquired resistance to metformin [221]. In this case, *Ppargc1a* expression facilitates metabolic flexibility where cells proliferated despite a reduced mitochondrial output and instead relying on an enhanced glycolysis flux. This response was a specific and transient adaptation to metformin exposure; when the drug was removed, Ppargc1a expression was reduced and the metabolism of the cells reverted to that of untreated cells.

PI3K inhibitors are another common metabolism-targeting treatment in cancer, effective by targeting the high glycolytic capacity of tumor cells. However, it has been shown that if tumor cells switch to metabolizing lactate instead of glucose, a phenomenon which has been shown in many cancer types [247], the cells evade the anti-proliferative effect of PI3K inhibitor and maintain tumor growth despite treatment [248]. Similarly, switching has also been shown for long term mTOR inhibition in lung squamous cell carcinomas. These tumors display a dependence on both glutamine and glycolytic metabolism - acute inhibition of either pathway had a negative effect on growth of these tumors. However, when glycolysis is inhibited long-term via prolonged administration of an mTOR inhibitor, the cells respond via GSK3 signaling and downstream Myc and cJUN by upregulating expression of glutaminase and thereby increasing glutamine catabolism [249]. Further evidence that cells can switch from relying on both glucose and glutamine to fuel the Krebs cycle pathways to just glutamine alone comes from a study in which glioblastoma cancer cells were prevented from channeling carbons from glucose into the mitochondria by pyruvate transporter inhibition. Here, a reduced entry of pyruvate into the mitochondria caused an increase in glutamate dehydrogenase activity to channel glutamine carbons into making oxaloacetate and acetyl-CoA [250].

Cancers can also adapt to nutrient restriction by upregulating pathways that allow cells to overcome this constraint. For example, Issaq et al. showed that when cell culture media is depleted of glutamine, following a lag (adaptive) period, sarcoma cells are able to proliferate through an upregulation of glutamine synthetase, which allows for *de novo* synthesis of glutamine, replacing the need for exogenous glutamine on which the cells once relied [251]. Parallel to this, it has been shown that, mediated by p53, an upregulation of an aspartate/glutamate transporter allows colon cancer cells to adapt to a glutamine free environment by maintaining oxidative phosphorylation through the utilization of aspartate as a substrate for *de novo* synthesis of glutamine and nucleotides [252].

Robustness of a tumor, in reference to metabolism, is also illustrated following angiogenesis-targeting therapeutics. Anti-angiogenic drugs often have an initial effect on a tumor caused by depletion of blood supply and resultant hypoxia. However, often times initial treatment success is followed by resistance, which can be conferred either by re-establishing pro-angiogenic pathways allowing the formation of new blood vessels, or by the tumor cells themselves acquiring resistance to low oxygenated environments. In the latter case, the tumor can grow despite a lack of blood supply [253]. A significant mechanism that enables tumor cells to do so, which has been shown in several tumor types, is the enhancement of glucose uptake and glycolytic flux, with a preference for the formation of lactate and diversion of carbons away from the Krebs cycle and oxidative phosphorylation, mediated by signaling pathways such as those regulated by HIF1a and mTOR [[254], [255], [256], [257], [258]]. This metabolic rewiring allows cells to cope with the new hostile environment induced by treatment and prevents tumor shrinkage even in the event of reduced angiogenesis.

Therefore, metabolic plasticity in tumors not only facilitates the transformation, growth, and spread of cancers but also allows tumors to survive treatment. Different drugs create different adaptive responses and will select for different populations within the tumor. A cell will strive to balance its metabolism in terms of energetic balance, supply of macromolecule precursors, and redox status, and the cells of a tumor are best placed to make this adaptation through clonal selection and direct reactive re-programming. Cancer cells will utilize all mechanisms possible to counteract inhibition of metabolic reactions induced by therapeutic regimes on the level of protein regulation, gene expression changes, and fast changes in the reactions that occur lead by the laws of chemistry. Consideration of the pre-existing metabolic differences between and within tumors and the ability of a tumor to metabolically adapt, e.g. by considering redundancy in metabolic pathways, will be key to designing successful treatment programs.

## 6 Conclusion and future outlook

There is no question that tumors are driven by underlying mutations, but the evolution of the tumor and the outgrowing clone will be determined largely by the pressure to cater for the tumor's metabolic needs. This is exacerbated by the fact that most major oncogenes heavily impinge on cellular metabolism and lock it into a proliferative state. Subsequently, tumors adapt to this new hyperproliferative state and, in doing so, diverge their metabolism from that found in normal tissues. This new metabolism, paired with the rapid growth of tumors that alters the micro-environment, typically creates a number of stress factors, such as hypoxia, nutrient paucity, and accumulation of metabolic waste products. These local stresses will further heighten the Darwinian pressure of clonal selection and ultimately yield tumors with a wide range of pathological metabolic phenotypes tailored to their respective niches. It is this metabolic specialization that is often regarded as the tumor's metabolic Achilles Heel and therapies targeting the reliance on certain pathways are steadily being added to our anti-tumoral arsenal.

However, to brand tumors as metabolically inflexible would be a gross oversight. Most obvious in the metastatic setting, the ability of tumor cells to dynamically adapt their metabolism in response to fluctuating stresses is a crucial feature of distant colonization. The symbiotic relationship between metabolic changes and EMT progression highlight the increased metabolic flexibility of cancer cells as the disease progresses. Metabolic adaptation is an absolute requirement for survival in environments of diverse nutrient and oxygen availability, which tumor cells encounter on their journey from the primary tumor to distal sites.

In many cases, tumors are able to further adapt to the therapeutically induced blockade of metabolic pathways and shift their metabolism to grow in the presence of a metabolic drug. Therefore, as a growing body of evidence shows, inhibition of multiple metabolic pathways is required to impede tumor expansion due to the inherent flexibility and redundancy in the structure of the metabolic network [[259], [260], [261], [262]].

## Funding

This work is supported by the Francis Crick Institute which receives its core funding from Cancer Research UK (FC001223), the UK Medical Research Council (FC001223), and the Wellcome Trust (FC001223) and by the CRUK Grand Challenge Award 2015 C57633/A25043.
